# Invasive intestinal mucormycosis in a 40-year old immunocompetent patient - a rarely reported clinical phenomenon: a case report

**DOI:** 10.1186/s12876-020-01202-5

**Published:** 2020-03-06

**Authors:** Abdi Bati Wotiye, Poornachandra KS, Biniyam Alemayehu Ayele

**Affiliations:** 1 Department of Gastroenterology and Hepatology, Fortis Hospital, Bannerghata Road, Bangalore, India; 2 Department of Internal Medicine, College of Medicine and Health Sciences, Hawassa University, Hawassa, Ethiopia; 3 Department of Neurology, College of Health Sciences, Addis Ababa University, PoBox 6396, Addis Ababa, Ethiopia

**Keywords:** Mucormycosis, Intestinal invasive, Immunocompetent

## Abstract

**Background:**

Mucormycosis is rare, life-threatening fungal infection. Frequently observed in those patients having underlying immunosuppression such as, diabetes, organ transplantation, Human immunodeficiency virus (HIV) infection, and elevated serum iron. However, invasive intestinal mucormycosis occurring in immunocompetent individuals without the traditional risk factors is extremely rare clinical phenomenon.

**Case presentation:**

We report a 40-year-old male patient who presented with 1 week history of diffuse abdominal pain and high grade fever, associated with vomiting and frequent loose stools. Has history of chronic alcohol ingestion. Otherwise, no past history of chronic medical illness, nor he had contact with individuals with similar illness. He was in a septic shock with multiple organ failure up on presentation to emergency room. Physical examination revealed icterus sclera with abdominal tenderness. He was immediately resuscitated using crystalloids, supported with inotrope, and antibiotics. Histopathological examination of tissue sample from colonic ulcer biopsy revealed invasive intestinal mucormycosis. Patient showed full clinical and histopathological resolution after course of parenteral Liposomal Amphotercin B.

**Conclusion:**

This case highlights the fact that, despite its life-threatening nature, it’s possible to treat patients with invasive intestinal mucormycosis with aggressive antifungal and supportive care without surgical intervention, provided that all the necessary supportive care were initiated early and the disease was diagnosed early and appropriate medical management was initiated timely. In addition, it’s important to consider intestinal mucormycosis even in patients who are immunocompetent without traditional risk factors.

## Background

Mucormycosis is an uncommon, life-threatening infection caused by fungi belonging to the subphylum Mucormycotina, order Mucorales. Among organisms responsible for causing mucormycosis, Rhizopus species are the most common cause of infection. The major portals of entry include the sinuses, lungs, gastrointestinal tract and skin [1, 2]. Mucormycosis mostly affects immunocompromised hosts including patients with diabetes, malignancy, HIV, organ transplant recipients and patients on immunosuppressive therapy [3]. While any organ system may be affected, rhino-orbital-cerebral and pulmonary infections dominate the literature [3–5]. Very few cases of gastrointestinal mucormycosis in an immunocompetent host have been reported [5, 6]. The aim of this case report is to highlight the importance of early detection and aggressive medical management of invasive mucormycosis may result in good outcome.

## Case presentation

This is a 40-year old man referred from local health facility to our hospital with diagnosis of acalculous cholecystitis after he presented with a week history of diffuse abdominal pain and high grade fever. Associated with this he had history of vomiting and frequent loose stools. He had history of chronic alcohol abuse. He has no urinary complaints, no past history of hypertension, diabetes mellitus, dyslipidemia or cardiac illness. No history of similar illness in the family and in his neighborhood. No history of contact with individuals suffering from similar illness, or traveling to region where such illness is common. He runs small business in the town. Physical examination showed emaciated but fully conscious. Vital signs were, Blood pressure = 80/50 mmHg, Pulse rate = 115/beat/minute, Temp = 99.6 °F, and RR = 26 breath/minute. Venous blood gas (VBG) analysis was suggestive of metabolic acidosis. He had scleral icterus bilaterally. In addition he had superficial and deep abdominal tenderness in epigastric and right upper quadrant region.

**Fig. 1:**
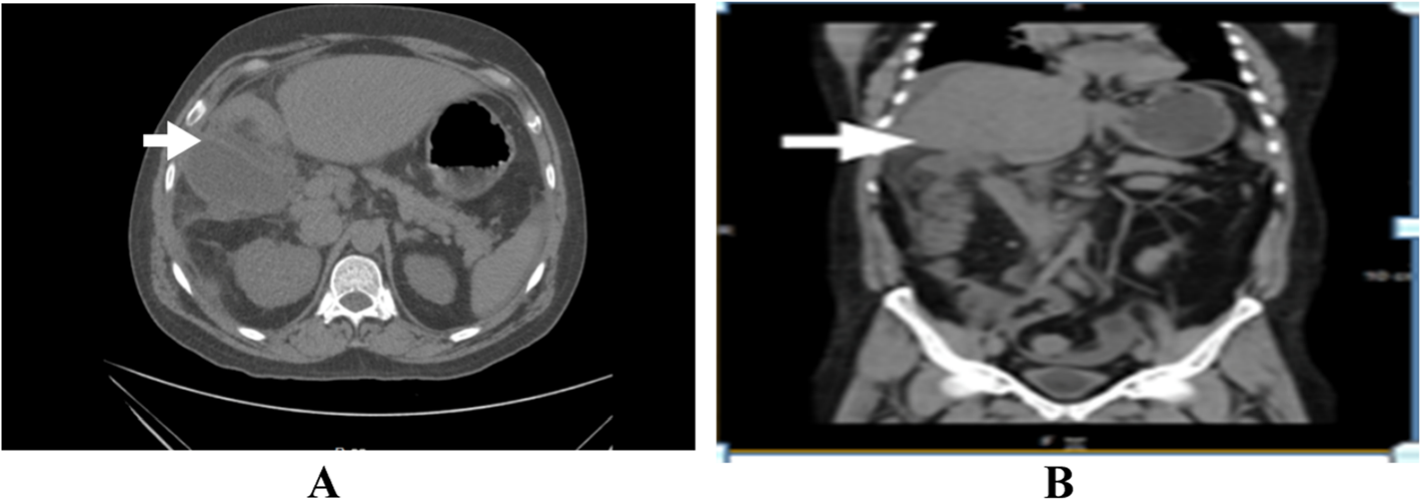
**a**, **b**:Axial and coronal CT scan of abdomen showing gall bladder wall edema with intense pericholecystic fat stranding (white arrow), free fluid around liver and showing edematous thickening (white arrow) at hepatic flexure

**Fig. 2:**
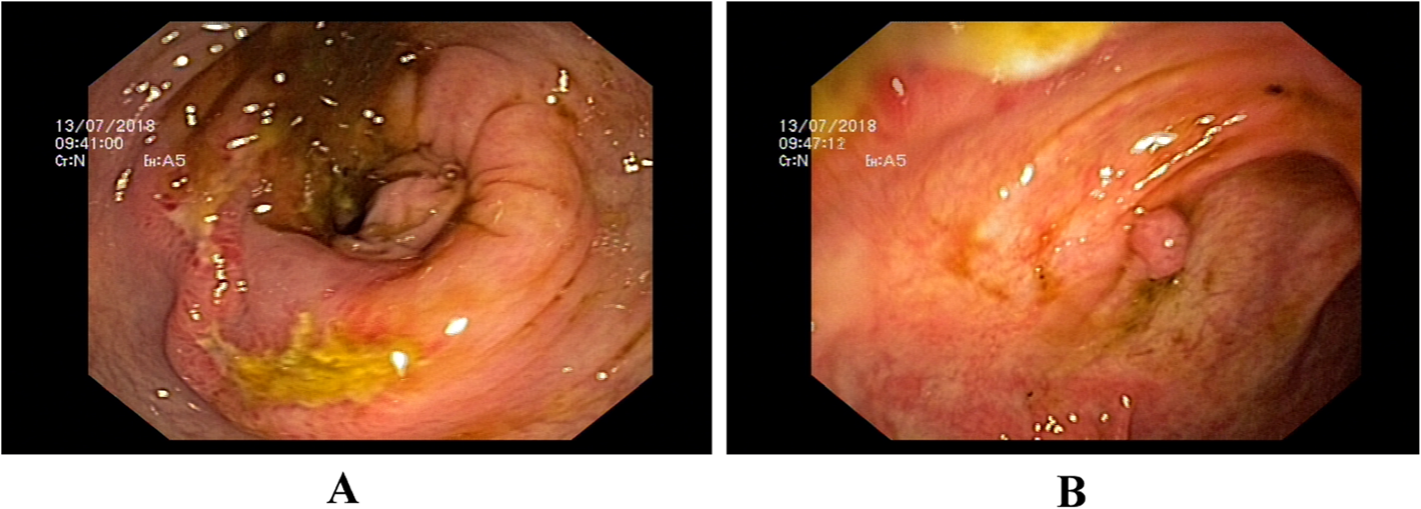
**a** Right transverse colon and hepatic flexure video colonoscopy image showing transverse ulcer with edematous mucosa. **b** showing a sessile polyp near the ulcer

Abdominal ultrasound showed mild hepatomegaly with mild course increased echo pattern, gall bladder wall thickening with mild pericholecystic fluid collection and mild ascites. Otherwise, the patient had no overt signs of liver cirrhosis. Computed tomography (CT) showed Gall bladder wall edema with intense pericholecystic fat stranding and free fluid around liver and spleen (Fig. 1a, b). There was mild ascites, nodular liver contours. The colon showed edematous thickening and ulcer at right transverse colon and hepatic flexure (Fig. 2a, b).

**Fig. 3:**
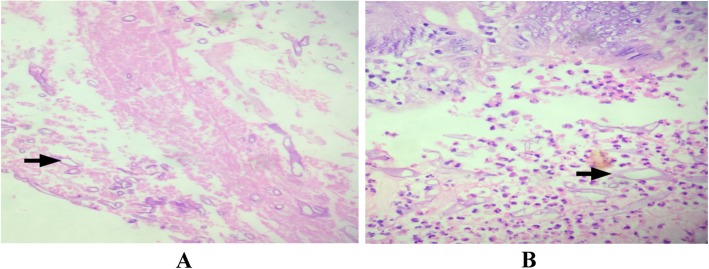
**a** Colon biopsies (hematoxylin and eosin (H&E) stain; 100x) from the center of a mucosal ulceration with sparsely septated hyphae of zygomycetes (black arrow) lying in fibrinous exudate. **b** Biopsy from a colon polyp with surface deposits of fungal filaments of zygomycetes (black arrow) lying in an acute suppurative inflammation exudate

**Fig. 4:**
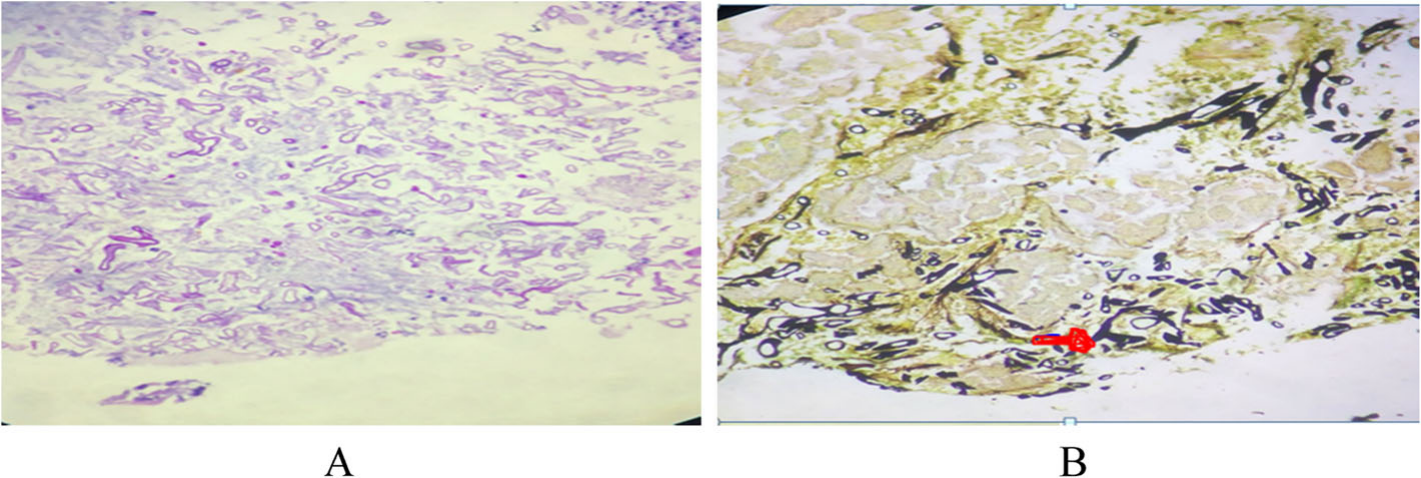
**a** & **b** Periodic acid-Schiff (PAS) stain and (B) Gomori Methenamine-Silver stain (GMS) highlighting the wide, ribbon-like, sparsely septated hyphae with wide-angle branching (approximately 90°; arrow), characteristic of zygomycetes species

He was admitted to medical ICU and started on parenteral antibiotics and inotropes and resuscitated with intravenous fluids. Urine and blood culture were negative. He was negative for Dengue IgM. On second day our patient’s condition was deteriorating despite broad spectrum antibiotics with persisting Fever, acidosis, patient become delirious, and lactate remained high (Table 1). Abdominal examinations showed increased tympanicity and diffuse tenderness. The patient was evaluated by GI surgeon and taken up for diagnostic laparotomy. Subsequently, mini laparotomy and cholecystotomy was done due to severe adhesions. Gall bladder fluid analysis was unremarkable, culture was negative, but histopathologic examination was not done as cholecystectomy was performed. On day 4, colonoscopy was done and sample was sent for histopathological analysis. Histopathology report revealed an acute suppurative inflammation of the colon mucosa with extensive areas of necrosis (Fig. 3a, b) and the detection of many wide, ribbon-like, sparsely septated fungal hyphae with wide-angle branching (approximately 90°), characteristic of zygomycetes species and thus consistent with mucormycosis (Fig. 4a, b). Microbiological culture and species subtyping was not done.

**Table 1:** Patient’s Laboratory test, results and references

Laboratory tests	Results	Reference range
WBC	11,700 cells/mm^3^ (N% 79)	4500–10,000 cells/mm^3^
Platelet count	116,000 cells/mm^3^	150,000 to 450,000 cells/mm^3^
Hemoglobin	13.3 g/dL	14.0 to 17.5 g/dL
Urea	71 mg/dL	4.3–22.4
Creatinine	2.29 mg/dL	5.1–14
SGOT	62 U/L	0–35 U/L
SGPT	19 U/L	0–35 U/L
Total bilirubin	3.14 mg/dL	0.3–1.0 mg/dL
Direct bilirubin	3.03 mg/dL	0.1–0.3 mg/dL
Alkaline phosphatase	110 U/L	30–120 U/L
GGT	60 U/L	9–50 U/L
Sodium	140 mEq/L	136–145 mEq/L
Potassium	3.87 mEq/L	3.5–5.0 mEq/L
Lactate	38 mmol/L	0.7–2.1 mmol/L
Fasting blood glucose	90 mg/dL	70–99 mg/dL
HIV 1/2, HBSAg and anti HCV		Negative

After diagnosis of invasive intestinal mucormycosis was confirmed by colonic biopsy results, antifungal medication with intravenous liposomal amphotericin B at a dose of 5 mg/kg daily was started on seventh day of admission. Lipid formulation of amphotericin B, rather than amphotericin B deoxycholate was selected, in order to deliver a high dose with less nephrotoxicity. Subsequently patient become afebrile and showed clinical improvement. Unfortunately, the patient developed acute kidney injury (Table 1) secondary to amphotericin B therapy, for which the drug was withdrawn for 2 days while the patient was kept received supportive care, latter antifungal treatment was resumed after 48 h. After receiving amphotericin B accumulative dose of 1.8 g patient clinical condition improved significantly. Finally he was discharged to local hospital to complete his total dose of 3.2 g of amphotericin B.

**Fig. 5:**
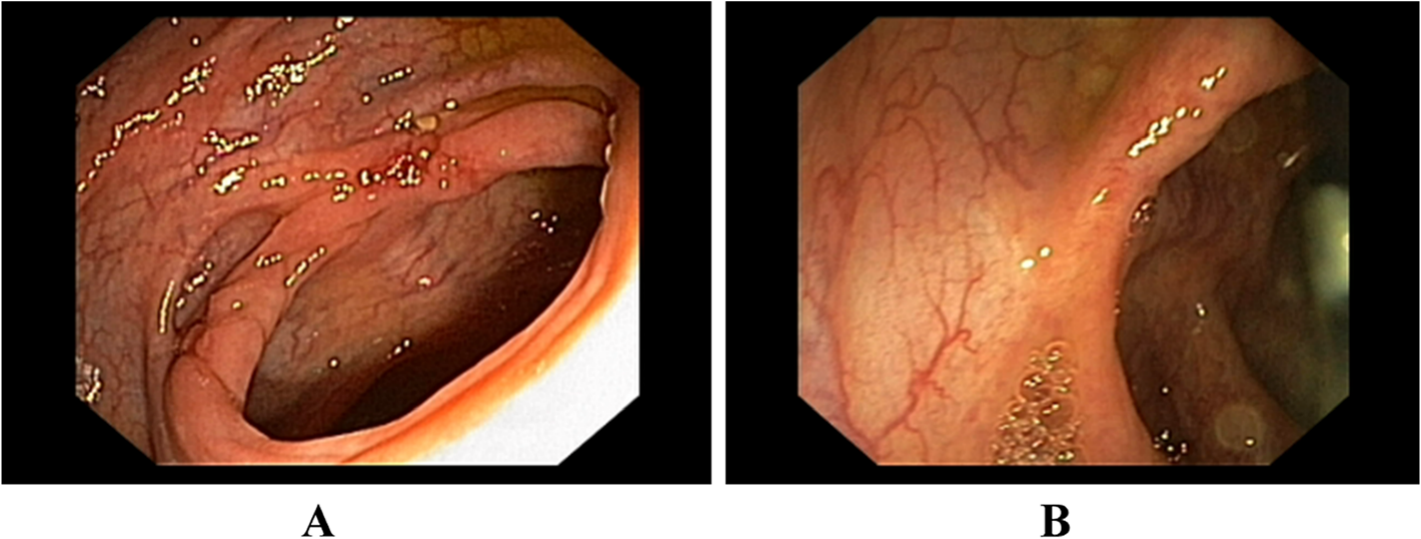
**a**, **b** Follow-up colonoscopy (At 2 weeks) showing healing ulcers in right transverse colon and hepatic flexure of the colon (**a**) and Follow-up colonoscopy at 6 weeks showing healed ulcer with scaring (**b**)

Even though intestinal mucormycosis carries high mortality even with treatment, our patient clinical manifestation improved significantly following antifungal treatment without surgical resection of necrotic tissue. This may highlight mucormycosis may have good prognosis if occurred in immunocompetent patients. Before his discharge the patient was screened for possible potential underlying predisposing factors such as diabetes mellitus, malignancy and HIV infection (Table 1), but he was negative for all. The only possible predisposing factor we identified was chronic alcoholism with high potential of liver cirrhosis. Patient was re-evaluated 2 weeks after his discharge and completion of his antifungal medication, he remained clinically stable and his liver and renal function tests were in normal range. Follow-up colonoscopy done showed healing hepatic flexure ulcers (Fig. 5a). Colonoscopy was repeated on 6 weeks follow up and showed healed hepatic flexure ulcers (Fig. 5b)**.**

## Discussion and conclusion

Mucormycosis is a life-threatening fungal infection caused by Mucorales, primarily affecting the immunocompromised hosts. Almost all patients with invasive mucormycosis have some underlying disease that both predisposes to the infection and influences the clinical presentation. However, it’s uncommon for immunocompetent patient to develop invasive life-threatening mucormycosis. Our case had no traditional risk factors often predisposing the patient to invasive mucormycosis. We only identified chronic alcoholic usage and early signs of liver cirrhosis as underlying risk factor in our patient [4, 7]. Despite the fact that invasive mucormycosis is lethal in more than half of the patient [1]**,** yet our patient showed excellent clinical responses to anti-fungal treatment alone without surgical resection of necrotic tissue. This benign course observed in our case is indicative of good outcome is possible if managed properly, especially in patient lacking traditional risk factors.

Involvement of gastrointestinal system by mucormycosis is dominated by involvement of the stomach in 67%, whereas the intestine is involved in 25% of the case. Intestinal mucormycosis has wide range of clinical manifestation, ranging from presenting with peptic ulcer disease to an aggressive and life threatening intestinal invasion by fungal filaments causing systemic fungemia [5, 6, 8]. The pathologic hallmark of mucormycosis is infarction of host tissue resulting from angioinvasion by fungal hyphae. This gives rise to necrotic ulcers with resultant acute abdominal pain, hematemesis, perforation and peritonitis. The patient is often thought to have an intra-abdominal abscess [1, 2]. Ante-mortem diagnosis of mucormycosis is made in only 25 to 50% of cases, as its extremely rare disease with significant fatal outcome even with treatment. Cultures are usually negative and no reliable serologic tests are currently available. The diagnosis is nearly always made by biopsy of the suspected area during or after surgery or endoscopy, or at autopsy [1, 2, 5, 9, 10].

Treatment of mucormycosis involves a combination of antifungal therapy with surgical debridement of infected and necrotic tissues [4–6, 10]. Additionally, early identification and treatment of an underlying predisposing factors, such as diabetes mellitus, immunosuppressive drugs, and neutropenia, chronic alcoholism, and HIV infection is vital to successfully treat invasive mucormycosis [5, 8, 11].

Gastro-intestinal mucormycosis has mortality rate of approximately 50% [1, 5, 10, 11]. Contrary to this fact, our patient has no identifiable traditional risk factors (Table 1), except history of chronic alcohol use. Furthermore, the patient was successfully treated without surgical resection with liposomal Amphotercin B further supporting the uniqueness of the case. Our patient received a total dose of 3.2grmas of Ampotercine B throughout the treatment course, in line with the recommended treatment regimen for such patients. Subsequently eradication of fungal infection was supported by normal follow up histopathological findings and normal follow-up colonoscopy examination.

Invasive intestinal mucormycosis is one of the rarest life-threatening fungal infection, because of this its vita to identify the illness early and initiate antifungal medication. Despite its high mortality our patient was salvaged because of the following critical measures: the patient was immediately admitted to medical ICU and resuscitation was initiated immediately using crystalloid fluid and inotropes. On top of this he was started on broad spectrum antibiotics in order to cover septicemia of pyogenic origin. In addition, the patient was re-evaluated frequently in order to detect any life threating complications, because of this he had abdominal CT and colonoscopy within few days of his admission and latter cholecystotomy was done to drain gall bladder collection. Finally histopathological results were made available in short period of time, which lead to initiation of antifungal drug.

Limitations to our approaches and management of this patient were: inability to remove the infected intestinal segment, lack of histopathological examination and biopsy gall bladder, even though patient was treated with antifungal, delayed diagnosis of mucormycosis is a serious and often fatal fungal infection and diagnosis is fraught with challenges due to its non-specific clinical features and need of invasive interventions. However, we report a case of immunocompetent patient with invasive intestinal mucormycosis being managed solely by parenteral antifungal medication without surgical resection and yet fully recovered from his illness after full course of antifungal treatment. Therefore, a high degree of clinical suspicion and early diagnosis helps in prompt antifungal therapy and improvement in survival.

## Data Availability

All data sets on which the conclusions of the case report based, to be available as spread sheets documents and available from the corresponding author on reasonable request from the editor.

## Abbreviations

AKI: Acute kidney injury
AMB: Amphotercin
BP: Blood pressure
CT: Computed tomography
ER: Emergency room
GGT: Gamma-glutamyltransferase
GMS: Gomori methenamine-silver
HCV: Hepatitis C virus
HIV: Human immunodeficiency virus
IV: Intravenous
LAMB: Liposomal Amphotercine B
^o^F: Degree fahrenheit
PAS: Periodic acid shift
SGOT: Aspartate aminotransferase
SGPT: Alanine aminotransferase
VBG: Venous blood gas
